# Supplementary material for: Assembly of the Tripartite and RNA Condensates of the Respiratory Syncytial Virus Factory Proteins In Vitro: Role of the Transcription Antiterminator M2-1
Source: Viruses. 2023 Jun 6;15(6):1329. doi: 10.3390/v15061329 (PMC10303025; doi:10.3390/v15061329)
Supplement: Supplementary file 1 [file viruses-15-01329-s001.zip › viruses-2422064-supplementary.pdf]

Supplementary materials

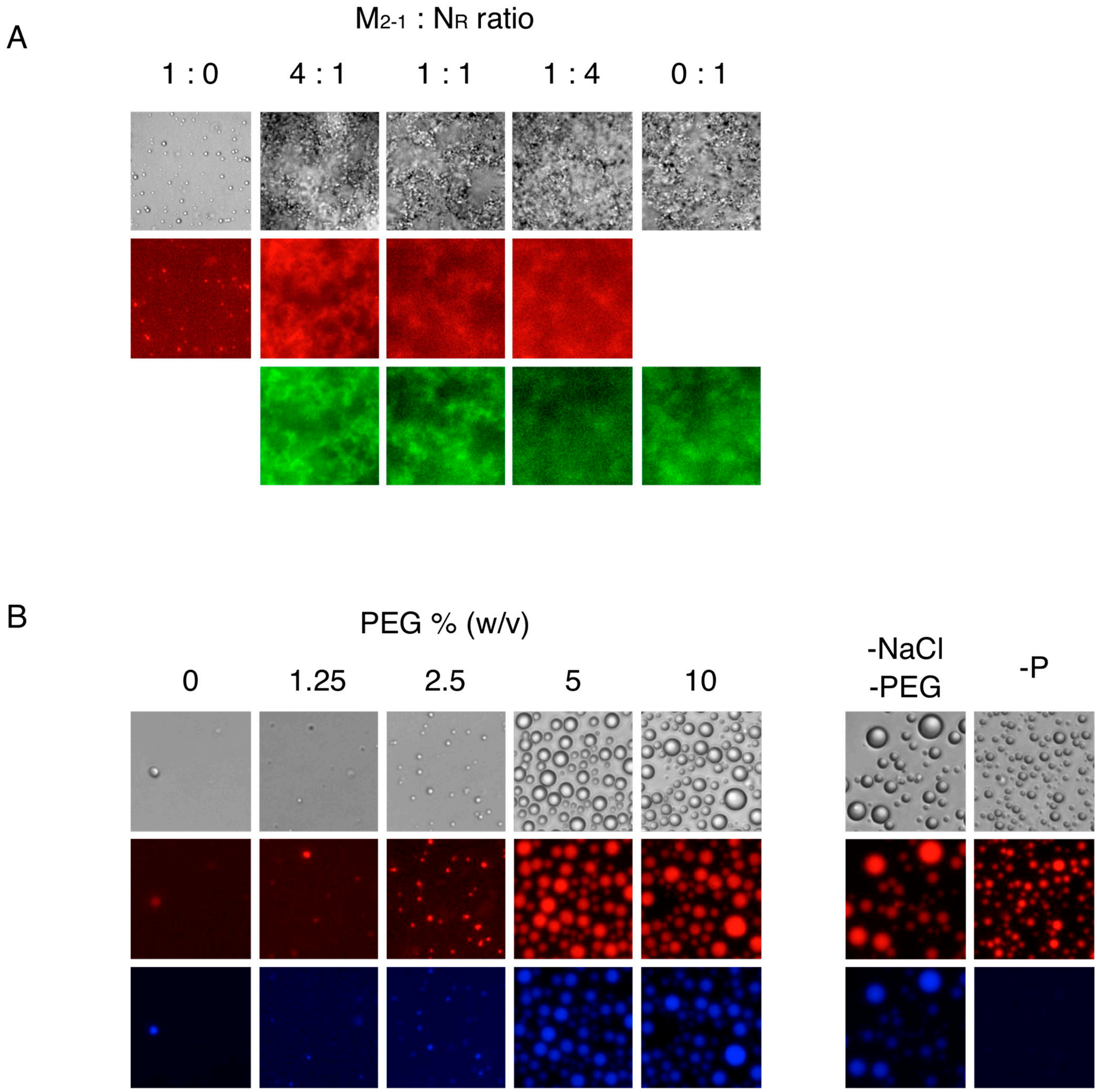

Figure S1. Heterotypic condensation of $M_{2\text{-}1}$ and P or N_R. (A) N_R and $M_{2\text{-}1}$ were incubated at different ratios with protein concentration ranging from 2.5 to 10 μM. (B) Effect of crowding agent on heterotypic LLPS. P and $M_{2\text{-}1}$ at 1:4 ratio were incubated in the presence of increasing concentrations of PEG-4000. -NaCl -PEG, corresponds to heterotypic P-$M_{2\text{-}1}$ condensates without NaCl and crowding agent. -P corresponds to homotypic $M_{2\text{-}1}$ condensates with fixed NaCl and PEG-4000.

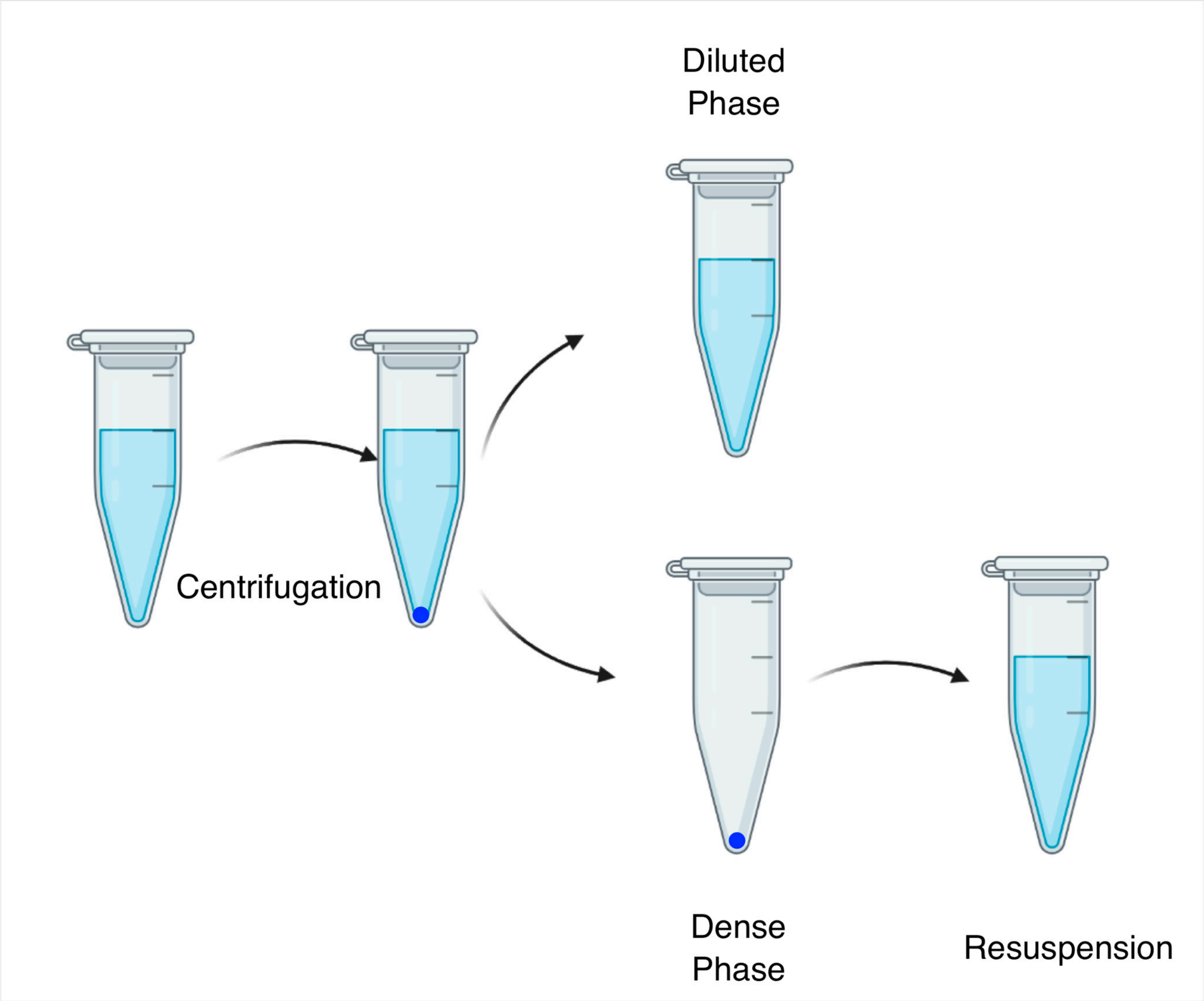

Figure S2. Scheme of partition experiment protocol.

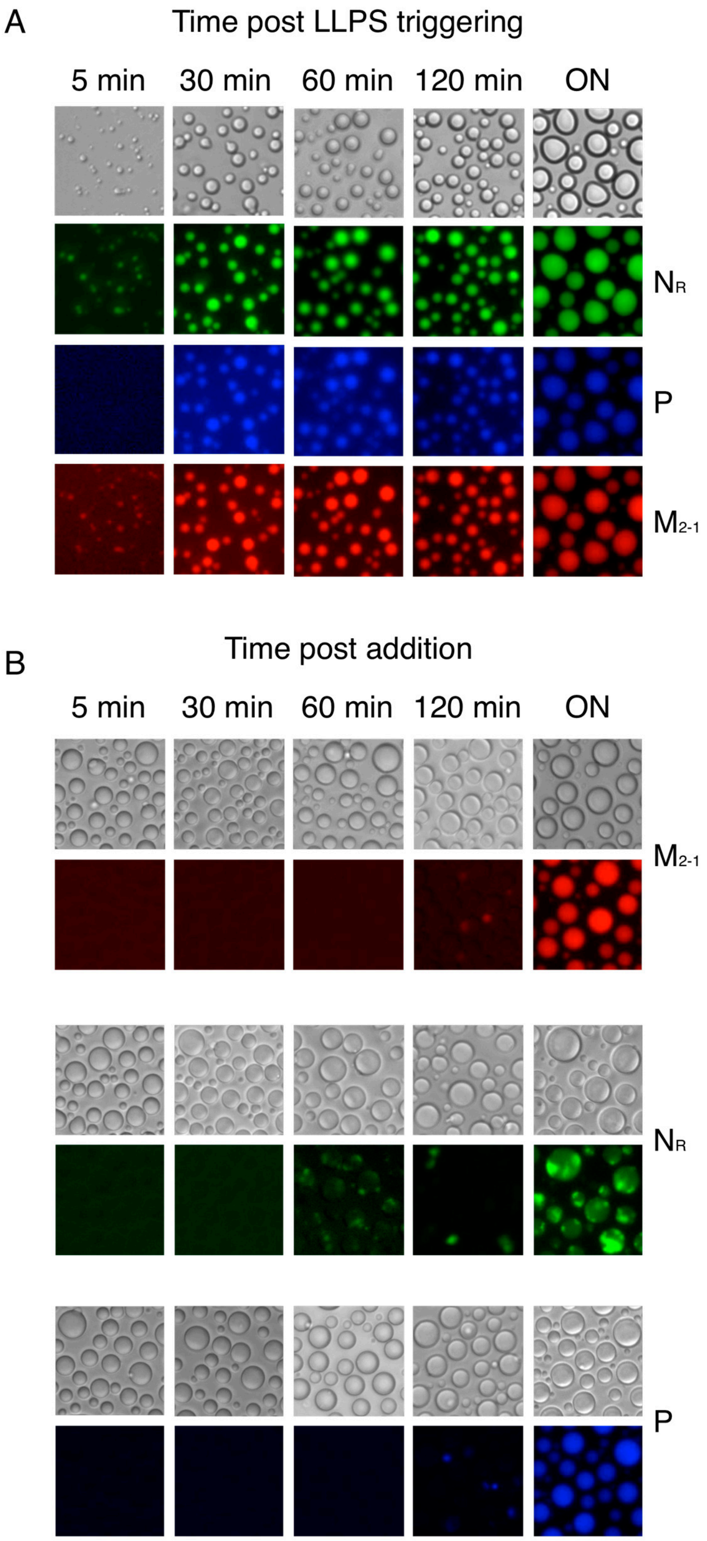

Figure S3. Tripartite P-N_R-M_{2-1} condensates. (A) Formation of tripartite heterotypic condensates triggered by N_R. Preformed P-M_{2-1} soluble complex incubated for 10 min and then N_R was added. (B) Incorporation of M_{2-1}-FITC, N_R-FITC or P-FITC to tripartite condensates of unlabeled P-N_R-M_{2-1}.

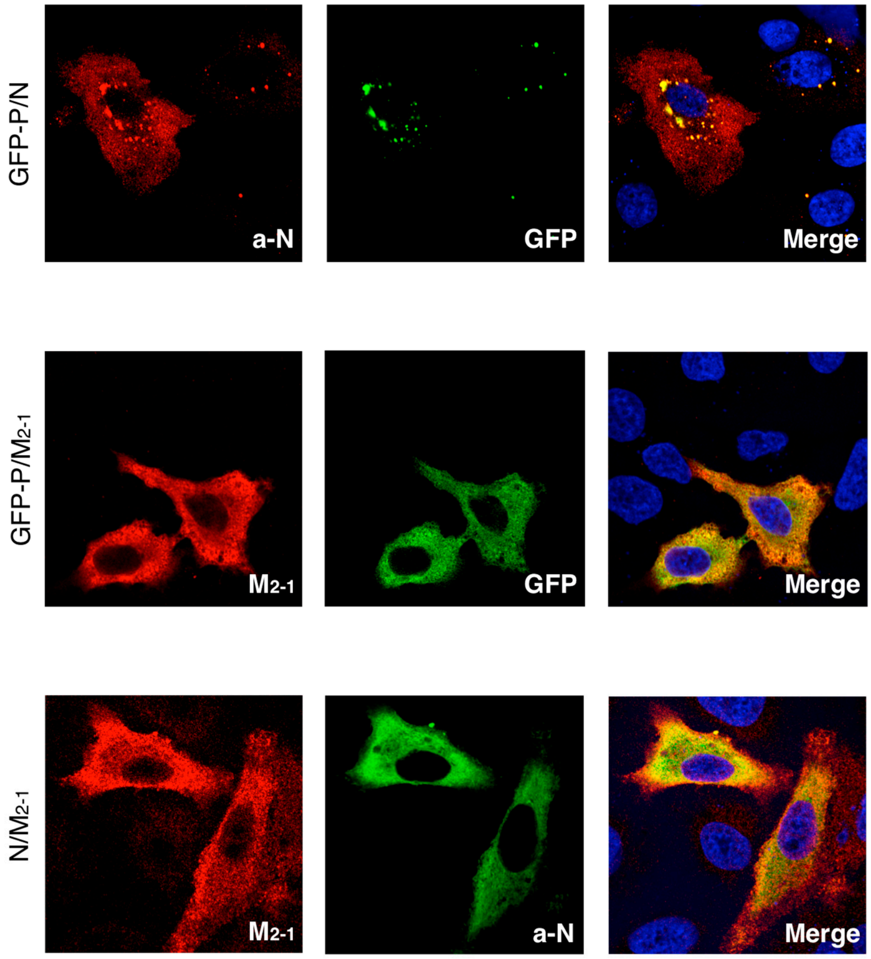

Figure S4. Co-localization of RSV proteins in transfected cells. A549 cells were co-transfected in pairs with plasmids encoding GFP-P, M_{2-1} and N for 24h as a control. Proteins were detected by fluorescence microscopy labeling by GFP fluorescence or with anti-M_{2-1} or anti-N antibodies. Nuclei were stained with DAPI.

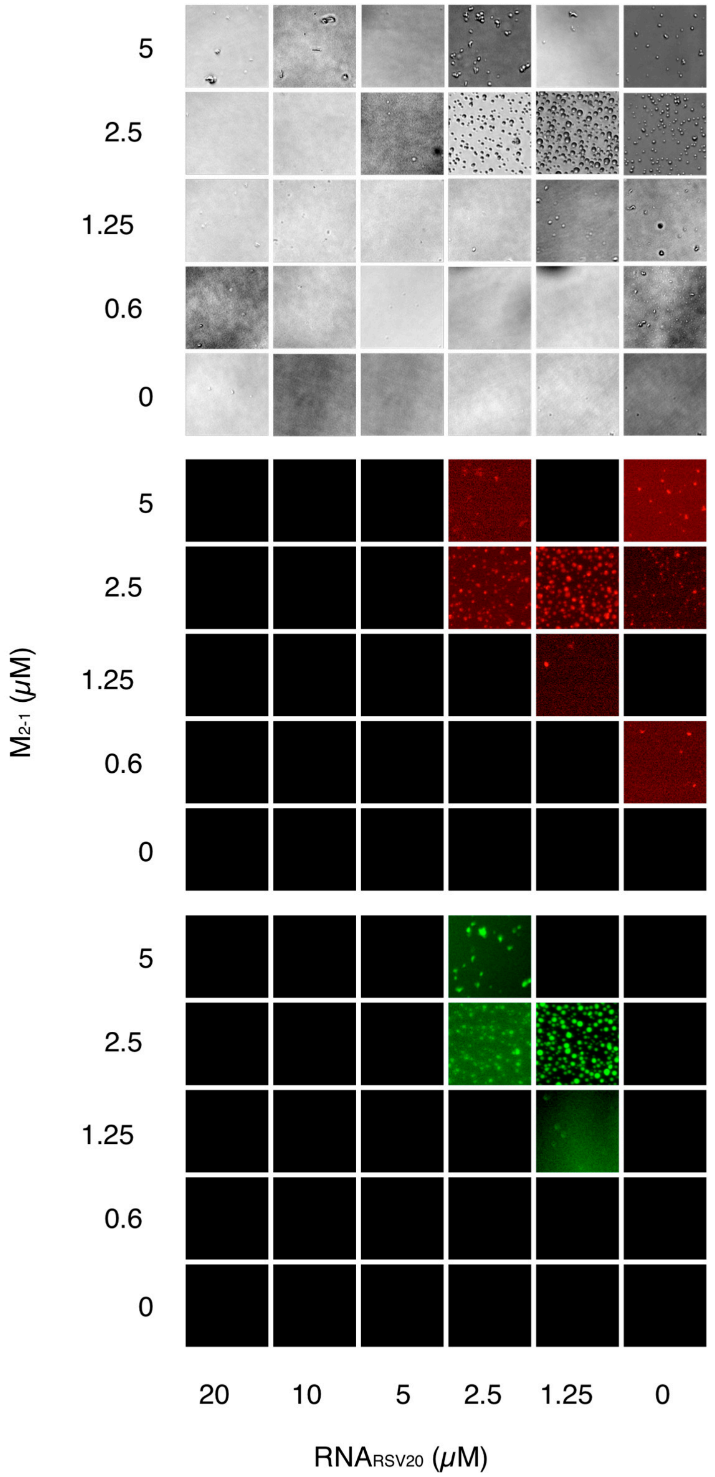

Figure S5. Grid of heterotypic M_{2-1}-RNA_{RSV20} condensates. Different concentrations of M_{2-1} and RSV_{RNA20} were visualized by brightfield microscopy (top) and fluorescence microscopy of M_{2-1}-Cy5 and RNA_{RSV20}-FITC (middle and bottom, respectively).

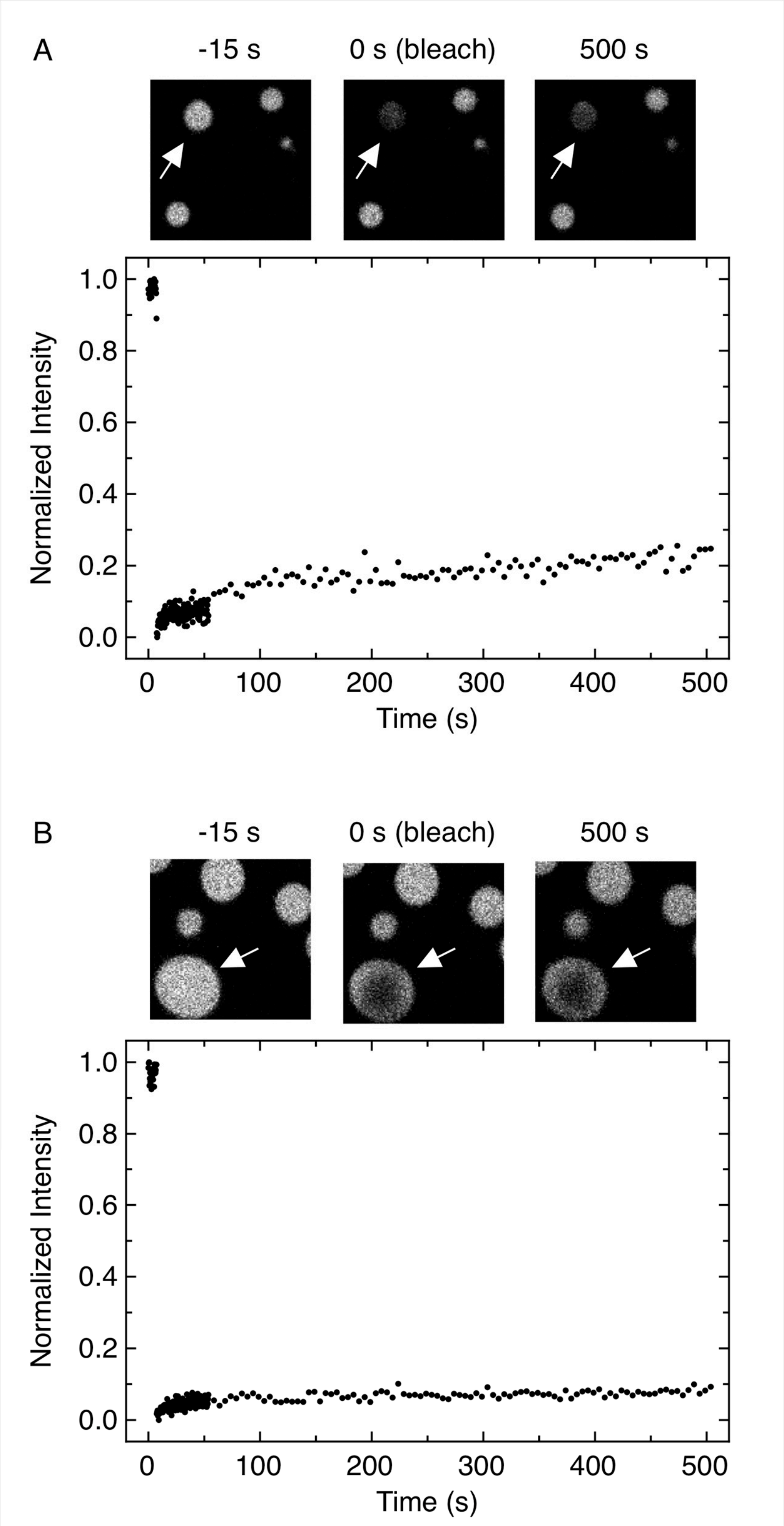

Figure S6. Fluorescence recovery after photobleaching (FRAP) assays of homotypic M_{2-1} and heterotypic M_{2-1}-tRNA$_{70}$ condensates. (A) Representative images and plot of FRAP analysis performed on FITC-M_{2-1} (5 μM) homotypic condensates. (B) Representative images and plot of FRAP analysis performed on FITC-M_{2-1} (5 μM) and tRNA$_{70}$ (300 mM) heterotypic condensates.

Table S1. Initial and final (condensed and diluted) protein concentration of heterotypic condensates after partition experiments.

	PEG-4000 (% w/v)	Concentration (μM)[1]		
		Initial mix	Dense phase	Diluted phase
P : M_{2-1} (4 : 1)	10	10 : 2.5	195 : 12	6.2 : 2.3
P : M_{2-1} (1 : 1)	10	2.5 : 2.5	53 : 24	1.5 : 2
P : M_{2-1} (1 : 4)	10	2.5 : 10	70: 140	1.1 : 7
P : N_R (2.5 : 1)	5	5 : 2	98.3 : 81.3	3 : 0.4
P : N_R : M_{2-1} (2.5 : 1 : 2.5)	5	5 : 5 : 2	120.92 : 93.38 : 64.58	2.6 : 0.1 : 3.7

[1] Refers to P tetramer, M_{2-1} tetramer and N_R decamer (see text).
